# Chronic obstructive pulmonary disease related to wood smoke and impact of the combined exposure to tobacco

**DOI:** 10.5588/ijtldopen.24.0004

**Published:** 2024-03-01

**Authors:** C.A. Torres-Duque, C. Jaramillo, A. Caballero, N.J. Proaños-Jurado, M.J. Pareja-Zabala, J.B. Soriano, M. González-García

**Affiliations:** ^1^ CINEUMO, Research Department, Fundación Neumológica Colombiana, Bogotá,; ^2^ Doctoral Biosciences, Universidad de La Sabana, Chía,; ^3^ Fundación Clínica Shaio, Bogotá,; ^4^ Clínica Reina Sofía, Bogotá,; ^5^ School of Medicine, Universidad de La Sabana, Chía, Colombia;; ^6^ Universitat de les Illes Balears, Palma de Mallorca, Spain

**Keywords:** biomass smoke, COPD, tobacco smoke, WS-COPD, TS-COPD, airflow obstruction

## Abstract

**BACKGROUND:**

Global Initiative for Chronic Obstructive Lung Disease (GOLD) 2023 highlights the need to explore aetiotypes of chronic obstructive pulmonary disease (COPD) beyond the tobacco-smoking COPD. Exposure to wood smoke (WS) is a risk factor for COPD in women, but the effect of the combined exposure to tobacco smoke (TS) in the general population and among COPD patients, and the characteristics of WS-COPD are unclear.

**METHOD:**

This was an analysis of data from PREPOCOL (Prevalence of COPD in Five Colombian Cities Situated at Low, Medium, and High Altitude), a random cross-sectional population-based study (*n* = 5,539) focusing on the effect of combined WS and TS exposure and WS-COPD characterisation.

**RESULTS:**

Prevalence of COPD was significantly higher in those exposed to both WS and TS (16.0%) than in those exposed to WS (6.7%) or TS (7.8%) only (*P* < 0.001). Exposure to WS was associated with COPD in men (OR 1.53, *P* = 0.017). WS-COPD individuals were more frequently female, older, shorter and had higher forced expiratory volume in 1 sec (FEV_1_) (all *P* < 0.05). Those exposed to both WS and TS had more symptoms and worse airflow limitation (*P* < 0.001).

**CONCLUSIONS:**

This was the first random population-based study showing that WS is an associated risk factor for COPD also in men, and that people exposed to both WS and TS have a significantly higher prevalence of COPD. Similarly, COPD subjects exposed to both types of smoke have more symptoms and greater airflow obstruction. This suggests an additive effect of WS and TS.

## RÉSUMÉ

**CONTEXTE:**

L'Initiative mondiale pour les maladies pulmonaires obstructives chroniques (Global Initiative for Chronic Obstructive Lung Disease, GOLD) 2023 met en évidence l'importance d'explorer les différents étiotypes de la maladie pulmonaire obstructive chronique (COPD, pour l’anglais « chronic obstructive pulmonary disease ») en dehors de la COPD liée au tabagisme. L'exposition à la fumée de bois (WS, pour l’anglais « wood smoke ») représente un facteur de risque de la COPD chez les femmes, cependant, l'impact de l'exposition simultanée à la fumée de tabac (TS, pour l’anglais « tobacco smoke ») chez la population générale et chez les patients atteints de COPD, ainsi que les caractéristiques spécifiques de la WS-COPD, demeurent peu clairs.

**MÉTHODES:**

Il s'agit d'une étude transversale aléatoire basée sur la population (n = 5 539) qui analyse les données de PREPOCOL (Prevalence of COPD in Five Colombian Cities Situated at Low, Medium, and High Altitude). L'étude se concentre sur l'effet de l'exposition combinée à la WS et à la TS ainsi que sur la caractérisation de la WS-COPD.

**RÉSULTATS:**

La prévalence de la COPD était significativement plus élevée chez les personnes exposées à la fois à la WS et à la TS (16,0%) que chez celles exposées uniquement à la WS (6,7%) ou à la TS (7,8%) (P < 0,001). L'exposition à la WS était associée à la COPD chez les hommes (OR 1,53 ; P = 0,017). Les personnes atteintes de WS-COPD étaient plus fréquemment des femmes, d'un âge plus avancé, de plus petite taille et présentaient un volume expiratoire maximal en 1 seconde (FEV1) plus élevé (tous P < 0,05). Les personnes exposées à la fois à la WS et à la TS ont montré plus de symptômes et une plus grande limitation du débit d'air (P < 0,001).

**CONCLUSION:**

Il s'agit de la première étude aléatoire basée sur la population qui démontre que la WS est un facteur de risque lié à la COPD, même chez les hommes, et que les individus exposés à la fois à la WS et à la TS présentent une prévalence significativement plus élevée de la COPD. De plus, les personnes souffrant de COPD qui sont exposés aux deux types de fumée manifestent davantage de symptômes et une obstruction pulmonaire plus sévère. Cela laisse supposer qu'il y a un effet cumulatif de la WS et de la TS.

Chronic obstructive pulmonary disease (COPD) remains a highly prevalent condition, causing significant morbidity and mortality worldwide.^1,2^ Recent publications, including the 2023 Report of the Global Strategy for Prevention, Diagnosis and Management of Chronic Obstructive Pulmonary Disease (GOLD), have highlighted the relevance of risk factors other than tobacco smoke (TS), and the need for a better characterisation and understanding of COPD aetiotypes other than the well-established TS-COPD.^2–4^ Although strong evidence remains insufficient, outdoor air pollution has been considered as a risk factor of COPD.^5,6^ On the other hand, long-term exposure to indoor air pollution derived from burning biomass fuels, including wood smoke (WS), has been related to COPD, chronic bronchitis (CB) and airflow obstruction in women,^7–10^ less consistently in men, and is currently accepted as a risk factor for COPD.^2,8^

An increasing number of studies have partially described the COPD related to biomass smoke, mainly of COPD associated with WS (WS-COPD), and have shown some significant differences of this condition with TS-COPD.^11–13^ In comparison with TS-COPD, WS-COPD occurs more frequently in women, mainly affects the airways with mild or no emphysema, and could have different pathogenetic mechanisms.^11,12,14–19^ However, knowledge about the prevalence and characteristics of patients with WS-COPD, and the effect of the combined exposure to WS and TS, is limited. Some studies have shown an increased risk of COPD and worse outcomes in smokers exposed to WS,^20,21^ as well as worse oxygen saturation in COPD patients exposed to both WS and TS;^16^ however, information about people chronically exposed to this combination, particularly from population-based studies, is scarce.

A better characterisation of biomass- and WS-COPD is necessary, not only because around 40% of the world population (about 2.8 billion people) continue to use solid fuels as household energy source,^22^ which has a large negative impact on global respiratory health,^23^ but because the treatment of biomass-related COPD could be different and its prevention could imply different approaches.

The PREPOCOL Study,^24^ aimed to determine COPD prevalence in Colombia, where exposure to WS was found to be a risk factor, offers a good opportunity to further explore the impact of combined WS and TS exposure on the general population and among COPD individuals, and the incompletely known characteristics of WS-COPD.

## METHODS

### Design and population

Information was obtained from the PREPOCOL Study,^24^ a random cross-sectional, population-based study conducted in urban areas of five Colombian cities. Detailed information about sample size and standardisation of measurements is provided in the original article.^24^ Subjects were selected using a probabilistic, two-stage clustered sampling technique. After providing informed consent, adults of both sexes, aged ≥40 years, who underwent high-quality spirometry and answered a respiratory questionnaire, were included. Demographic, socio-economic, clinical and spirometry variables were collected. The study and informed consent were approved by the Institutional Research Ethics Committee (*Comité de Ética en Investigación de la Fundación Neumológica Colombiana*) Bogotá, Colombia..

### Questionnaire and spirometry

We used a Spanish version of the Standardized Respiratory Questionnaire for Epidemiologic Studies of the American Thoracic Society (ATS-DLD-78A) with the following additional questions on WS exposure: Have you ever used wood for cooking habitually? If yes, for how many years? What type of fuel do you currently use for cooking? (Including wood as an option). Habitual use was defined as most days of the week. Pre-/post-bronchodilator (BD) spirometry (MicroLoop; Micro Medical, Rochester, UK) was performed according to ATS/ERS (European Respiratory Society) recommendations. Crapo et al.’s reference values were used.^25^

### Definitions

COPD was defined by a post-BD ratio of forced expiratory volume in 1 sec (FEV_1_) to forced vital capacity (FVC) of <0.70,^2^ and CB as an affirmative answer to the question: Have you ever had cough and expectoration for three or more months a year for at least two consecutive years? Cough and phlegm were considered present if an affirmative answer was obtained for the following questions, respectively: 1) Do you usually have cough? 2) Do you usually bring up phlegm from your chest, not from the back of your nose? Usually was defined as most days of the week.

### Group categorisation according to exposure to wood or tobacco smoke

All participants (*n* = 5,539) and subjects with COPD (*n* = 494) were categorised in four groups: 1) WS group: exposed to WS ≥10 years and to TS <10 packs/year; 2) TS group: exposed to TS ≥10 packs/year and to WS <10 years; 3) combined group (MS): exposed to both WS ≥10 years and to TS ≥10 packs/year; and 4) not exposed to either WS or TS. The cut-off point for WS exposure (≥10 years as risk factor of COPD) was based on the previously published multivariate analysis in the same population;^24^ The cut-off for TS of 10 packs/year is reasonably supported for discriminating the risk of COPD.^26^ For comparative analyses among COPD individuals, we focused on the three exposed groups; the non-exposed group was excluded (*n* = 35, 7.1%).

### Statistical analysis

Our analysis addressed COPD prevalence and factors associated with COPD and CB in the entire population. A logistic regression model was constructed using the variables that showed *P* < 0.1 in the univariate analysis. Odds ratios (ORs) for COPD were estimated according to exposure to WS, TS or both using non-exposed people as reference, and adjusting by sex, age, educational level, city of residence, self-reported history of TB and occupational exposure to vapours, gases, dust, and fumes (VGDF). After the first analysis, we focused on individuals with COPD to compare the following clinical and functional characteristics of three groups according to exposure to WS, TS or both (combined): age, sex, educational level, height, body mass index (BMI), symptoms (cough, phlegm, dyspnoea, wheezing), FVC, FEV_1_ and FEV_1_/FVC. As recommended by Halbert et al., we grouped the participants into two categories for age-specific estimates: ≥40–64 and >64 years.^27^ Differences between COPD groups were evaluated using the χ^2^ test and analysis of variance. *P* < 0.05 was considered for statistical significance. Interactions between WS and TS exposures were explored. The statistical software SigmaStat v3.2 (Informer Technologies, Los Angeles, CA, USA) was used.

## RESULTS

### Prevalence and associated factors according to exposures in the entire population

A total of 5,539 participants were included. Table 1 shows the distribution of the entire study population and the people with COPD. In comparison with non-COPD, participants with COPD were older, more frequently male, exposed to both WS and TS, and were more likely to have a history of TB and occupational exposure (all *P* < 0.001). Of the study population, 60% was exposed to WS: 30.9% (*n* = 1,713) to WS only and 29.8% (*n* = 1,650) to both WS and TS (combined). There were no significant differences in exposures between the cities.

**Table 1.** Demographic and clinical characteristics of participants.

Variable	Total	Non-COPD	COPD	*P* value
	(*n* = 5,539)	(*n* = 5,045, 91.1%)	(*n* = 494, 8.9%)	
	*n* (%)	*n* (%)	*n* (%)	
City of residence				<0.001
Bogotá	1,106 (20.0)	1,012 (20,1)	94 (19.0)	
Bucaramanga	1,103 (19.9)	1,016 (20,1)	87 (17.6)	
Cali	1,100 (19.9)	1,007 (20,0)	93 (18.8)	
Barranquilla	1,102 (19.9)	1,034 (20,5)	68 (13.8)	
Medellín	1,128 (20.4)	976 (19.3)	152 (30.8)	
Age, years*				<0.001
<64	4,108 (74.2)	3,903 (77.4)	205 (41.5)	
≥64	1,431 (25.8)	1,142 (22.6)	289 (58.5)	
Sex				<0.001
Female	3,701 (66.8)	3,457 (68.5)	244 (49.4)	
Male	1,838 (33.2)	1,588 (31.5)	250 (50.6)	
Education level				<0.001
Secondary or higher	1,752 (31.6)	1,665 (33.0)	87 (17.6)	
Primary or none	3,786 (68.4)	3,379 (67.0)	407 (82.4)	
WS and TS exposure				<0.001
WS^†^	1,713 (30.9)	1,599 (31.7)	114 (23.1)	
TS^‡^	1,035 (18.7)	954 (18.9)	81 (16.4)	
Combined WS and TS^§^	1,651 (29.8)	1,387 (27.5)	264 (53.4)	
No WS or TS	1,140 (20.6)	1,105 (21.9)	35 (7.1)	
Self-report history of TB				<0.001
No	5,477 (98.9)	4,999 (99.1)	478 (96.8)	
Yes	62 (1.1)	46 (0.9)	16 (3.2)	
Occupational exposure to VGDF				<0.001
No	3,129 (56.5)	2,890 (57.3)	239 (48.4)	
Yes	2,410 (43.5)	2,155 (42.7)	255 (51.6)	

* Age grouping as recommended by Halbert et al.^26^

^†^ Exposed to WS ≥10 years and to TS <10 packs/year. Supporting evidence for exposure cut-off points is provided in the Methods section.

^‡^ Exposed to TS ≥10 packs/year and to WS <10 years. Supporting evidence for exposure cut-off points is provided in the Methods section.

^§^ Exposed to both WS ≥10 years and to TS ≥10 packs/year. Supporting evidence for exposure cut-off points is provided in the Methods section.

COPD = chronic obstructive pulmonary disease; WS = wood smoke; TS = tobacco smoke; VGDF = vapours, gases, dust, and fumes.

The overall prevalence of COPD was 8.9% (*n* = 494). According to exposures, the COPD prevalence was significantly higher in the combined group (i.e., exposed to both WS and TS) (16.0%) than in the TS group (7.8%) or the WS group (6.7%) (*P* < 0.001). Similarly, the prevalence of CB was higher in the MS group (9.0%) than in the TS group (5.2%) or the WS group (4.2%) (*P* < 0.001).

After adjusting for age, sex, smoking, educational level, occupational exposures and history of TB, exposure to WS ≥10 years was associated with COPD in both women (OR 1.84, 95% CI 1.31–2.60; *P* < 0.001) and men (OR 1.53, 95% CI 1.08–2.18; *P* = 0.017). COPD prevalence increased significantly according to the duration of exposure to WS, reaching 23.2% in those exposed ≥30 years (Figure). The unadjusted and adjusted ORs for COPD were significantly higher in the MS group than in the WS or TS groups (Table 2). No statistical interactions between WS and TS exposures were identified.

**Figure.**
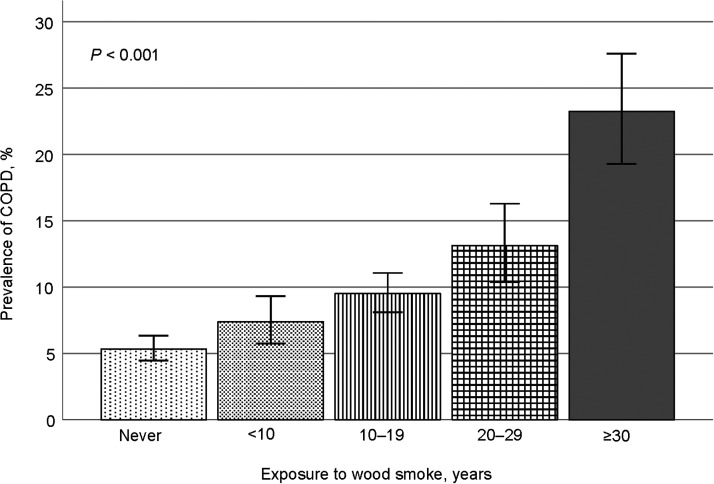
Prevalence of COPD according to length of wood smoke exposure (*n* = 5,539). COPD = chronic obstructive pulmonary disease.

**Table 2.** Unadjusted and adjusted ORs for COPD (airflow obstruction) by exposure (*n* = 5,539).

Exposure	Unadjusted OR (95% CI)	*P* value	aOR (95% CI)*	*P* value
WS^†^	2.25 (1.53–3.31)	<0.001	1.61 (1.08–2.40)	0.021
TS^‡^	2.68 (1.79–4.02)	<0.001	2.10 (1.38–3.18)	0.001
Combined WS and TS (MS-COPD)^§^	6.01 (4.19–8.62)	<0.001	2.99 (2.04–4.38)	<0.001
No WS or TS exposure (reference group)	1.0		1.0	

* Adjusted by sex, age, educational level, city of residence, self-reported history of TB and occupational exposure to vapours, gases, dust, and fumes.

^†^ Exposed to WS ≥10 years and to TS <10 packs/year. Supporting evidence for exposure cut-off points is provided in the Methods section.

^‡^ Exposed to TS ≥10 packs/year and to WS <10 years. Supporting evidence for exposure cut-off points is provided in the Methods section.

^§^ Exposed to both WS ≥10 years and to TS ≥10 packs/year. Supporting evidence for exposure cut-off points is provided in the Methods section.

OR = odds ratio; COPD = chronic obstructive pulmonary disease; CI = confidence interval; aOR = adjusted OR; WS = wood smoke; TS = tobacco smoke.

### Characteristics of COPD individuals according to exposure

Of the 494 participants with COPD, 35 (7.1%) had no history of exposure to either WS or TS and these individuals were not considered for the comparative analyses. Table 3 presents the demographic, clinical and spirometry characteristics of the 459 participants with COPD (47.8% of whom were women) according to exposure to WS, TS or both (combined). Those with WS-COPD were predominantly female and had significantly lower height and higher BMI than those with TS-COPD (*P* < 0.05). Women exposed to WS (WS-COPD and combined) were older (*P* = 0.036) and referred more frequently to dyspnoea (*P* = 0.004); they also had lower height and higher BMI than women with TS-COPD (*P* < 0.001). Educational level was significant lower in people with COPD exposed to WS (WS-COPD and combined) than in those with TS-COPD (*P* < 0.001).

**Table 3.** Demographic, clinical and spirometric characteristics in COPD groups by exposure (*n* = 459).

	WS-COPD*	TS-COPD^†^	MS-COPD, WS+TS^‡^	*P* value
	(*n* = 114)	(*n* = 81)	(*n* = 264)	
	Mean ± SD	Mean ± SD	Mean ± SD	
Female sex, *n* (%)	90 (78.9)^§¶^	32 (39.5)	97 (36.7)	<0.001
Age, years	64.3 ± 11.1	61.5 ± 10.9^#^	66.9 ± 10.7	<0.001
Weight, kg	61.3 ± 12.5	63.2 ± 13.3	63.3 ± 13.1	0.342
Height, cm	154.1 ± 8.5^§¶^	162.3 ± 8.1	159.6 ± 9.5	<0.001
BMI, kg/m^2^	25.8 ± 4.8^§^	23.9 ± 4.0	24.9 ± 4.8	0.026
Education level, secondary or higher	15 (13.2)^§^	34 (42.0)^#^	23 (8.7)	<0.001
Persistent cough, *n* (%)	19 (16.7)^¶^	21 (25.9)	81 (30.7)	0.018
Persistent phlegm, *n* (%)	19 (16.7)^¶^	16 (19.8)^#^	90 (34.1)	0.001
Dyspnoea, *n* (%)	60 (52.6)	36 (44.4)	150 (56.8)	0.144
FVC post-BD, %pred	95.5 ± 19.8	95.5 ± 18.7	92.4 ± 18.1	0.204
FEV_1_ post-BD, %pred	76.9 ± 19.0^¶^	73.9 ± 18.4	71.7 ± 19.3	0.048
FEV_1_/FVC post-BD, %	63.8 ± 6.3^¶^	61.2 ± 7.9	60.5 ± 9.2	0.002

* Exposed to WS ≥10 years and to TS <10 packs/year.

^†^ Exposed to TS ≥10 packs/year and to WS <10 years.

^‡^ Exposed to both WS ≥10 years and to TS ≥10 packs/year.

Evidence support for exposure cut-off points is provided in the Methods section.

^§^ *P* < 0.05 between WS-COPD and TS-COPD.

^¶^ *P* < 0.05 between WS-COPD and MS-COPD.

^#^ *P* < 0.05 between TS-COPD and MS-COPD.

COPD = chronic obstructive pulmonary disease; WS = wood smoke; SD = standard deviation; MS = mixed smoke (i.e., WS + TS); TS = tobacco smoke; BMI = body mass index; FVC = forced vital capacity, BD = bronchodilator. FEV_1_ = forced expiratory volume in 1 sec.

### Combined exposure to wood and tobacco smoke in COPD

Of 494 individuals with COPD, 264 (53.4%) were exposed to both WS and TS (combined [MS group]). In the MS or combined group, exposures to WS (median: 17.0 years, interquartile range [IQR] 10.0–30.0) and to TS (median: 21.0 packs/years, IQR 8.8–34.5) were not significantly different from those of the WS group (median: 19.0 years, IQR 12.0–30.0; *P* = 0.174) and the TS group (median: 25.5 packs/years, IQR 17.6–41.5; *P* = 0.091). Individuals in the combined group were more likely to have persistent cough (*P* = 0.018) and phlegm (*P* = 0.001), and had significantly lower post-BD FEV_1_% and FEV_1_/FVC% (*P* = 0.002), both in the total group and in women only (*P* = 0.003) than in the WS or TS groups (Table 3).

## DISCUSSION

This study not only demonstrates that exposure to WS for ≥10 years is a contributing factor to COPD in women but also establishes, for the first time in a random population-based study, that this exposure is also associated with COPD in men. Moreover, individuals exposed to both WS and tobacco smoke (TS) exhibit a significantly higher prevalence of COPD and chronic bronchitis (CB) than those exposed to either WS or TS alone. Furthermore, our study innovatively showed that people with COPD exposed to both WS and TS are more likely to have persistent respiratory symptoms (cough and phlegm) and significantly greater airflow limitation (lower post-BD FEV_1_% and FEV_1_/FVC%) than those exposed to WS or TS. Finally, we found that people and women with WS-COPD, as a biomass COPD type, have different characteristics than those with TS-COPD: they are older and have significantly shorter height, higher BMI and lower educational level.

These findings in individuals with combined exposure to WS and TS, both in the general population (higher prevalence and associations with COPD and CB) and in individuals with COPD (worse clinical and functional outcomes) suggest an additive adverse effect of combined exposure. Although the questionnaires used do not allow us to define the precise sequence of the combined exposure by length of exposure, combined exposure was simultaneous in many of our study participants.

Although the information about the effect of the combined exposure to biomass smoke (i.e., WS + TS) is still scarce, some study findings are in line with our results.^20,21^ In a smokers cohort, Sood et al. found that self-reported WS exposure was independently associated with lower FEV_1_ and a higher prevalence of airflow obstruction and CB.^20^ In the Lovelace Smokers Cohort, individuals with WS exposure experienced a more rapid decline of FEV_1_ and worse quality of life than those without WS exposure.^21^ Recently, Olloquequi et al. described significantly lower oxygen saturation in patients with COPD and combined exposure to biomass smoke and TS.^16^ López-Campos et al. found that patients with one other factor in addition to TS exposure had more chronic sputum production, worse scores in the COPD Assessment Test and greater long-term oxygen therapy requirement.^28^

Our findings suggest confluent pathophysiologic mechanisms due to combined exposure. Sood et al. showed that WS exposure interacted with aberrant promoter methylation of the *p16* or *GATA4* genes, increasing the risk of COPD and lower FEV_1_ in smokers.^20^ Awji et al. found that WS enhances TS-induced inflammation in airway epithelial cells.^29^ The inflammatory pathways induced by biomass smoke/WS or TS could be different,^16,19,30,31^ and could interact in enhancing the negative effects of the combined exposure.

As previously described,^11–13,32,33^ we found that people with WS-COPD are predominantly female and older, and have significantly shorter height and higher BMI than TS-COPD. Cooking in developing countries is traditionally done by women who also spend more time indoors. Here, for the first time in a random population-based study, we show that exposure to WS ≥10 years was associated with COPD in men as well, probably due to poor household ventilation and the proximity of the kitchen, leading to prolonged exposure to high concentrations of pollutants from WS throughout the home. This finding is similar to that previously described in a meta-analysis.^34^

The older age observed in patients with WS-COPD suggests a different pattern of exposure–response than in those with TS-COPD,^11^ whereby exposure over a longer period of time is probably required for the development of COPD. Similarly, adjusted by age, airflow obstruction was milder in those with WS-COPD as described in other studies,^11,12,29,32,33^ suggesting also a lower decline of FEV_1_.^33^ However, no clear explanation for the lower height and higher BMI in the general population and in women exposed to WS (both WS-COPD and in the combined group) than in those with TS-COPD could be ascertained. The use of biomass fuels for cooking is associated with low socio-economic status; it is possible that the low birth weight of children, which is linked to maternal prenatal exposure to biomass fuels,^35^ is associated with reduced height in adulthood. In addition, although we did not collect data on the race/ethnic origins of the participants, in Colombia, indigenous ethnicity is more frequent in people from rural areas, who are more likely to be exposed to biomass fuels than people from urban areas, where White and mixed races are more frequent. Andean Colombian indigenous groups have lower height and higher BMI than the general Colombian population.^36^

The higher frequency of cough, phlegm and CB in those with WS-COPD than in those with TS-COPD has been described.^12,18^ The novelty of this study lies in its revelation that the occurrence of these symptoms, particularly dyspnoea in women, is further elevated among those with concurrent exposure to both WS and TS. In addition, this is the first study to show that COPD individuals exposed to both WS and TS have greater airflow obstruction (lower FEV_1_ and lower FEV_1_/FVC) than those exposed to either WS or TS. These observations might indicate the cumulative effects of extensive airway damage caused by WS, such as bronchial anthracofibrosis,^12,15–18,35,37^ along with damage induced by TS in the airways and lungs, suggesting interacting pathophysiological mechanisms.^19–21,28^ It is not clear if the results of our study can be extrapolated to COPD caused by other types of biomass fuels.

Our study strengths include its randomised design, and a large and representative number of participants in total and in each of the groups of interest according to smoke exposure: wood, tobacco and combined. The large number of participants identified with COPD, both on the whole and in each group, allowed us to reliably analyse associations, characterise groups and accurately describe differences. There were also some limitations, mainly due to the cross-sectional design of the study; as information was based on questionnaire responses about past exposures and events, we were unable to confirm causality but only suggest associations. Data on the intensity of WS exposure (ventilation conditions, proximity of the kitchen and type of stove used) were lacking. Nevertheless, questions about the length of exposure and type of fuel routinely used for cooking make the analysis and conclusions robust.

GOLD 2023^2^ and COPD Lancet Commission^3^ have highlighted the relevance of risk factors for COPD other than TS, and the need for a better characterisation and understanding of these aetiotypes. Our study presents important information about a type of biomass COPD due to indoor air pollution derived from WS and the impact of the combined exposure to WS and TS, and highlights the need for research on the underlying mechanisms.

## References

[1] Soriano JB, .; GBD 2015 Chronic Respiratory Disease Collaborators. Global, regional, and national deaths, prevalence, disability-adjusted life years, and years lived with disability for chronic obstructive pulmonary disease and asthma, 1990–2015: a systematic analysis for the Global Burden of Disease Study 2015. Lancet Respir Med 2017;5:691–706. 28822787 10.1016/S2213-2600(17)30293-X PMC5573769

[2] Global Initiative for Chronic Obstructive Lung Disease. Global strategy for prevention, diagnosis and management of COPD: 2024 Report. https://goldcopd.org/2024-gold-report/ Accessed on January 2024.

[3] Stolz D, . Towards the elimination of chronic obstructive pulmonary disease: a Lancet Commission. Lancet 2022;400:921–972. 36075255 10.1016/S0140-6736(22)01273-9 PMC11260396

[4] Celli B, . Definition and nomenclature of chronic obstructive pulmonary disease: time for its revision. Am J Respir Crit Care Med 2022;206:1317–1325. 35914087 10.1164/rccm.202204-0671PP PMC9746870

[5] Wang L, . Air pollution and risk of chronic obstructed pulmonary disease:The modifying effect of genetic susceptibility and lifestyle. EBioMedicine 2022;79:103994. 35417845 10.1016/j.ebiom.2022.103994 PMC9018147

[6] Thurston GD, . Outdoor air pollution and new-onset airway disease. an official American Thoracic Society Workshop Report. Ann Am Thorac Soc 2020;17:387–398. 32233861 10.1513/AnnalsATS.202001-046ST PMC7175976

[7] Sana A, . Chronic obstructive pulmonary disease associated with biomass fuel use in women:a systematic review and meta-analysis. BMJ Open Respir Res 2018;5:e000246. 10.1136/bmjresp-2017-000246 PMC5786909 29387422

[8] Pathak U, Gupta NC, Suri JC. Risk of COPD due to indoor air pollution from biomass cooking fuel:a systematic review and meta-analysis. Int J Environ Health Res 2020;30:75–88. 30754998 10.1080/09603123.2019.1575951

[9] Kurmi OP, . COPD and chronic bronchitis risk of indoor air pollution from solid fuel: a systematic review and meta-analysis. Thorax 2010;65:221–228. 20335290 10.1136/thx.2009.124644

[10] Gonzalez-Garcia M, . Chronic bronchitis:High prevalence in never smokers and underdiagnosis–a population-based study in Colombia. Chron Respir Dis 2019;16:1479972318769771. 29669432 10.1177/1479972318769771 PMC6302977

[11] Perez-Padilla R, Ramirez-Venegas A, Sansores-Martinez R. Clinical characteristics of patients with biomass smoke-associated COPD and chronic bronchitis. Chronic Obstr Pulm Dis 2014;1:23–32. 28848808 10.15326/jcopdf.1.1.2013.0004 PMC5559138

[12] Torres-Duque CA, Garcia-Rodriguez MC, Gonzalez-Garcia M. Is chronic obstructive pulmonary disease caused by wood smoke a different phenotype or a different entity? Arch Bronconeumol 2016;52:425–431. 27207325 10.1016/j.arbres.2016.04.004

[13] Assad NA, . Chronic obstructive pulmonary disease secondary to household air pollution. Semin Respir Crit Care Med 2015;36:408–421. 26024348 10.1055/s-0035-1554846

[14] Camp PG, . COPD phenotypes in biomass smoke- versus tobacco smoke-exposed Mexican women. Eur Respir J 2014;43:725–734. 24114962 10.1183/09031936.00206112

[15] González-García M, . Tomographic and functional findings in severe COPD:comparison between the wood smoke-related and smoking-related disease. J Bras Pneumol 2013;39:147–154. 23670499 10.1590/S1806-37132013000200005 PMC4075823

[16] Olloquequi J, . Comparative analysis of COPD associated with tobacco smoking, biomass smoke exposure or both. Respir Res 2018;19:13. 29347936 10.1186/s12931-018-0718-y PMC5774164

[17] Zhao D, . Small airway disease:A different phenotype of early stage COPD associated with biomass smoke exposure. Respirology 2018;23:198–205. 28906034 10.1111/resp.13176

[18] Ramirez-Venegas A, . Small airway disease in COPD associated to biomass exposure. Rev Invest Clin 2019;71:70–78. 30810542 10.24875/RIC.18002652

[19] Ortiz-Quintero B, Martínez-Espinosa I, Pérez-Padilla R. Mechanisms of lung damage and development of COPD due to household biomass-smoke exposure:inflammation, oxidative stress, microRNAs, and gene polymorphisms. Cells 2023;12:67. 10.3390/cells12010067 PMC9818405 36611860

[20] Sood A, . Wood smoke exposure and gene promoter methylation are associated with increased risk for COPD in smokers. Am J Respir Crit Care Med 2010;182:1098–1104. 20595226 10.1164/rccm.201002-0222OC PMC3001253

[21] Leng S, . Wood smoke exposure affects lung aging, quality of life, and all-cause mortality in New Mexican smokers. Respir Res 2022;23:236. 36076291 10.1186/s12931-022-02162-y PMC9454202

[22] Stoner O, . Household cooking fuel estimates at global and country level for 1990 to 2030. Nature Comm 2021;12:5793. 10.1038/s41467-021-26036-x PMC8490351 34608147

[23] Soriano JB, Soriano C, Fernandez E. Respiratory planetary medicine. Arch Bronconeumol 2017;53:297–299. 27979636 10.1016/j.arbres.2016.10.016

[24] Caballero A, . Prevalence of COPD in five Colombian cities situated at low, medium, and high altitude (PREPOCOL study). Chest 2008;133:343–349. 17951621 10.1378/chest.07-1361

[25] Crapo RO, Morris AH, Gardner RM. Reference spirometric values using techniques and equipment that meet ATS recommendations. Am Rev Respir Dis 1981;123:659–664. 7271065 10.1164/arrd.1981.123.6.659

[26] Chang JT, . Prediction of COPD risk accounting for time-varying smoking exposures. PLoS One 2021;16:e0248535. 33690706 10.1371/journal.pone.0248535 PMC7946316

[27] Halbert RJ, . Global burden of COPD:systematic review and meta-analysis. Eur Respir J 2006;28:523–532. 16611654 10.1183/09031936.06.00124605

[28] Lopez-Campos JL, . Occupational and biomass exposure in chronic obstructive pulmonary disease:results of a cross-sectional analysis of the On-Sint Study. Arch Bronconeumol 2017;53:7–12. 27432162 10.1016/j.arbres.2016.04.013

[29] Awji EG, . Wood smoke enhances cigarette smoke-induced inflammation by inducing the aryl hydrocarbon receptor repressor in airway epithelial cells. Am J Respir Cell Mol Biol 2015;52:377–386. 25137396 10.1165/rcmb.2014-0142OC PMC4370262

[30] Silva R, Oyarzun M, Olloquequi J. Pathogenic mechanisms in chronic obstructive pulmonary disease due to biomass smoke exposure. Arch Bronconeumol 2015;51:285–292. 25614376 10.1016/j.arbres.2014.10.005

[31] Golpe R, . Differences in systemic inflammation between cigarette and biomass smoke-induced COPD. Int J Chron Obstruct Pulmon Dis 2017;12:2639–2646. 28979110 10.2147/COPD.S141068 PMC5589102

[32] Golpe R, . Distribution of clinical phenotypes in patients with chronic obstructive pulmonary disease caused by biomass and tobacco smoke. Arch Bronconeumol 2014;50:318–324. 24576449 10.1016/j.arbres.2013.12.013

[33] Ramírez-Venegas A, . The “slow horse racing effect” on lung function in adult life in chronic obstructive pulmonary disease associated to biomass exposure. Front Med (Lausanne) 2021;8:700836. 34307427 10.3389/fmed.2021.700836 PMC8295605

[34] Hu G, . Risk of COPD from exposure to biomass smoke:a metaanalysis. Chest 2010;138:20–31. 20139228 10.1378/chest.08-2114

[35] Pope DP, . Risk of low birth weight and stillbirth associated with indoor air pollution from solid fuel use in developing countries. Epidemiol Rev 2010;32:70–81. 20378629 10.1093/epirev/mxq005

[36] Meisel A, Vega M. La estatura de los colombianos: un ensayo de antropometría histórica, 1910–2002. Bogota, Colombia: Banco de La República, 2004.

[37] Kim YJ, Jung CY, Shin HW, Lee BK. Biomass smoke induced bronchial anthracofibrosis: presenting features and clinical course. Respir Med 2009;103:757–765. 19111453 10.1016/j.rmed.2008.11.011

